# Coccidiosis: Recent Progress in Host Immunity and Alternatives to Antibiotic Strategies

**DOI:** 10.3390/vaccines10020215

**Published:** 2022-01-29

**Authors:** Youngsub Lee, Mingmin Lu, Hyun S. Lillehoj

**Affiliations:** Animal Biosciences and Biotechnology Laboratory, United States Department of Agriculture, Agricultural Research Service, Beltsville, MD 20705, USA; youngsub.lee@usda.gov (Y.L.); mingmin.lu@usda.gov (M.L.)

**Keywords:** coccidiosis, *Eimeria*, poultry, immunity, vaccine, alternatives to antibiotics

## Abstract

Coccidiosis is an avian intestinal disease caused by several distinct species of *Eimeria* parasites that damage the host’s intestinal system, resulting in poor nutrition absorption, reduced growth, and often death. Increasing evidence from recent studies indicates that immune-based strategies such as the use of recombinant vaccines and various dietary immunomodulating feed additives can improve host defense against intracellular parasitism and reduce intestinal damage due to inflammatory responses induced by parasites. Therefore, a comprehensive understanding of the complex interactions between the host immune system, gut microbiota, enteroendocrine system, and parasites that contribute to the outcome of coccidiosis is necessary to develop logical strategies to control coccidiosis in the post-antibiotic era. Most important for vaccine development is the need to understand the protective role of the local intestinal immune response and the identification of various effector molecules which mediate anti-coccidial activity against intracellular parasites. This review summarizes the current understanding of the host immune response to coccidiosis in poultry and discusses various non-antibiotic strategies which are being developed for coccidiosis control. A better understanding of the basic immunobiology of pertinent host–parasite interactions in avian coccidiosis will facilitate the development of effective anti-*Eimeria* strategies to mitigate the negative effects of coccidiosis.

## 1. Introduction

Coccidiosis is a major enteric infection of poultry that is estimated to cost more than USD 14.5 billion annual losses globally [1]. Although coccidiosis control using various anticoccidial chemicals, such as ionophores, coccidiocides, and coccidiostats, has long been a mainstream strategy in modern poultry production, alternative control strategies to antibiotics are necessary owing to the antibiotic ban [2]. Therefore, much effort has been made to develop alternative strategies, including vaccines and dietary strategies using phytochemicals, probiotics, prebiotics, hyperimmune antibodies, and bacteriophages. Since the 1950s, the control of coccidiosis in commercial chicken production has been successful using live or attenuated vaccines [3]. However, live vaccines require a host to replicate parasites or to induce active immunity, which is a fundamental disadvantage as this requires a considerable amount of time and money, and there is a high risk of inducing subclinical coccidiosis. In addition, in the absence of growth promoters, live vaccines can increase the incidence of bacterial enteritis [4]. 

In response to these concerns, the demand for cost-effective and safe anticoccidial vaccines is rapidly increasing. Immunological approaches can provide an alternative method to prevent the spread of coccidiosis while avoiding the shortcomings of conventional measures. Immunologic approaches include vaccination with recombinant vaccines, immunostimulation with cytokines, and antibiotic alternatives to improve host innate immunity using antimicrobial host peptides such as NK-lysin [5]. However, a detailed understanding of the *Eimeria* lifecycle, intestinal immune response, and the intricate interaction between parasites and the gut microbiome is required for the effective application of new immunotherapeutics including vaccines in commercial practice. Therefore, this review will provide the current knowledge on the host immune response to coccidiosis in poultry and discuss the efficacy of various *Eimeria* vaccine candidate antigens in protecting chickens against coccidiosis. Moreover, alternative countermeasures such as hyperimmune antibodies, antimicrobial peptides, prebiotics, and probiotics will be discussed. 

## 2. Coccidiosis in Chickens

Coccidiosis is caused by pathogenic *Eimeria* species and there are seven different *Eimeria* spp. (*E. acervulina*, *E. brunetti*, *E. maxima*, *E. mitis*, *E. necatrix*, *E. praecox*, and *E. tenella*) that undergo intracellular development in a site-specific manner. Each *Eimeria* spp. infects a specific region of the intestine, for example, *E. tenella* infects caecum whereas *E. acervulina* targets duodenum [6]. Among the seven *Eimeria* spp., *E. necatrix*, and *E. tenella* are classified as highly pathogenic in poultry [7], but all seven species of *Eimeria* infect the gastrointestinal tract which is the primary organ responsible for digestion and nutrient absorption. Therefore, coccidiosis results in serious economic consequences due to inefficient feed utilization, impaired growth rate, high mortality, and a temporary reduction in egg production. In addition, infection with certain *Eimeria* spp., especially *E. maxima,* is the primary risk factor for necrotic enteritis since coccidiosis compromises gut integrity allowing the proliferation of toxinogenic *Clostridium perfringens* [8,9,10]. 

Coccidiosis is initiated upon the ingestion of sporulated oocysts that contain four sporocysts by chickens. As oocysts travel down the gastrointestinal track, physical grinding and/or gut digestive enzymes break the oocyst wall to release four sporocysts with two sporozoites each per sporulated oocyst. Sporozoite is the invasive form of *Eimeria* and, through an unknown mechanism, it invades specific sites in the gut epithelium to undergo first intracellular development and release merozoites through the merogony cycle by asexual reproduction. One sporozoite produces approximately 1000 merozoites and this process can be repeated two to four times. After the asexual lifecycle, the gametogony stage of the sexual reproduction cycle begins, during which male and female gametes form. Fertilization of the male and female gametes results in the formation of zygotes encased by thick outer walls which develop into unsporulated oocysts that shed onto the litter in the feces. *Eimeria* parasites show high host-specificity, although no information is available on the mechanism of host specificity. Many factors such as genetics, sex, nutrition, biochemistry, and immunity play a role and interaction between these factors dictates the outcome of coccidiosis [11]. Each successive cycle exponentially increases the number of oocysts in the environment. Therefore, unless anticoccidials are used or blocking immunity has developed, naive chickens cannot cope with this sudden, massive exposure to infective sporulated oocysts. These observations and the understanding of the *Eimeria* life cycle suggest distinct antigenic diversity among *Eimeria* spp., which is essential for vaccine development. Although most infectious diseases affecting the poultry industry have been effectively controlled using various methods [9,12,13], coccidiosis remains the most unconquerable disease in poultry fields because of its resistance to climatic change and the ability of *Eimeria* parasites to retain their infectivity for a long time. The use of vaccines is considered the most practical and safe method to control coccidiosis in poultry, and many types of vaccines using whole parasites, either live or attenuated, have been developed.

## 3. Host Immune Response to Coccidiosis

### 3.1. Role of Gut-Associated Lymphoid Tissues (GALT) and Mucosal-Associated Lymphoid Tissues (MALT)

The most fundamental function of the gut is to digest food through masticatory movement and absorb nutrients into the bloodstream. Thus, the intestinal epithelium is constantly in contact with resident microorganisms and food and is constantly exposed to a wide range of potential pathogenic microorganisms. The mucosal immune system is composed of mucosal-associated lymphoid tissues (MALTs) of the mammary glands, genital tract, respiratory tract, and intestine [14]. Among these, gut-associated lymphoid tissues (GALTs) represent the largest compartment of the immune system, and more than half of GALT is composed of MALT. These mucosal surfaces protect the body from an enormous quantity and variety of harmful antigens, and the primary role of GALT is to prevent progression to systemic infection by detecting and destroying infectious agents at an early stage. 

The MALT of chickens is well developed [15], and the first line of defense against various pathogenic antigens starts with MALT. Since *Eimeria* is also an intestinal parasite, the first line of defense against coccidiosis starts in GALT. GALT serves three major functions in host defense against coccidia infection: antigen processing and immunogenic epitope presentation, the release of intestinal antibodies, and the activation of cell-mediated immunity [16]. GALT is a multilayered tissue comprising an outer epithelial cell and a row of lymphocytes above the basement membrane. Immediately below the basement membrane, the lamina propria contains lymphocytes and the submucosa. In chickens, various specialized lymphoid organs such as Peyer’s patches, the bursa of Fabricius, and cecal tonsils which contain various immune cells (e.g., epithelial, lymphoid, natural killer cells, dendritic cells, and antigen-presenting cells), have evolved to protect the host from invading pathogens [17,18]. Host immune response is highly regulated in GALT through complex mechanisms, including cytokine secretion, lymphocyte stimulation, and the activation of resident immune cells [19]. Antigen recognition and immune activation mainly occur at the site of GALT involving Peyer’s patches of lamina propria [16]. GALT contains B and T lymphocytes, which play a critical role in acquired immunity against avian coccidiosis [20]. 

The gastrointestinal epithelium is mostly covered with a protective mucus gel composed of glycoproteins produced by goblet cells, and this mucous layer acts as a physical barrier against pathogen invasion [21]. Furthermore, other factors have been observed in the intestinal tract, such as lysozymes, microbial flora, gastric secretion, bile salts, and endogenous cationic peptides, that also function as non-specific barriers [20]. Gallinacin is a chicken epithelial defensin which is predominantly expressed in the tongue, the bursa of Fabricius, and the trachea of normal chickens, and protects against microbial invasion [22]. Defensin is an essential peptide for host defense that provides immediate protection against bacterial invasion. However, the exact role of defensin in local defense against coccidial infections has not been well studied. GALT routinely encounters not only numerous pathogens but also nonpathogenic microbes and self-antigens. Therefore, an improved understanding of avian GALT is important to develop oral vaccines, antibiotic alternative feed additives, or potential anti-inflammatory compounds to maintain gut homeostasis during infections [23].

### 3.2. Role of Cell-Mediated Immune Response 

Adaptive immunity includes humoral and cell-mediated immune (CMI) responses, which are mediated by soluble antibodies and T lymphocytes, respectively. Both responses play important roles against extracellular and intracellular antigens, with CMI response predominantly involved in host defense against intracellular pathogens.

The CMI response against intracellular antigens mainly targets exogenous antigens that have entered cells via the endocytic pathway or endogenous antigens produced within the cell, such as viral proteins and residues that result from the neoplastic transformation of the cell [24]. T cells play the most important role in response to primary or challenge coccidia infection [25]. Most humoral immune responses and the CMI response are regulated by various subpopulations of T lymphocytes that express different repertoires of T-cell receptors (TCRs) capable of recognizing multiple antigens [24]. As in mammals, there are two main types of T cells in chickens: CD4^+^ (cluster of differentiation 4^+^) helper T cells (TH) and CD8^+^ cytotoxic T cells (TC). Mammals and chickens are critically dependent on CD4^+^ TH cells in adaptive immunity [26]. The activation of T cells is determined by major histocompatibility complex (MHC) antigens, whereby cytotoxic T lymphocytes recognize foreign antigens in association with MHC class I molecules and T helper cells recognize antigens in the context of MHC class II molecules [27]. In this process, co-stimulatory signals are essential for full activation of T cell [28]. 

Initial studies have found that T cells play a crucial role in mediating anti-coccidial immunity in chickens [29]. Many cytotoxic T lymphocytes expressing CD8 cell surface antigens have been observed in primary *Eimeria*-infected chickens [30,31]. In addition, Rose et al. [32] demonstrated that CD4^+^ and CD8^+^ T lymphocytes contribute differently to primary and secondary *Eimeria* infections. Following coccidiosis, increasing amounts of T cells secreting interferon (IFN)-γ [33], an immunoregulatory cytokine, appear, and these activate the proinflammatory pathway and inhibit intracellular *Eimeria* parasite development in cells [34]. Flow cytometric analysis of the intestinal epithelial lymphocytes (IELs) of naive and *Eimeria*-infected chickens using lymphocyte-specific immune reagents demonstrated the relevance of different T cell subpopulations in mediating the anticoccidial innate immune response after exposure to *Eimeria* infection [35]. Since circulating effector memory T cells are recruited into intestinal epithelium where *Eimeria* parasites undergo intracellular development [36], the role of various effector lymphocyte populations in the gut and their role in developing resistance against secondary coccidiosis need further study [37]. In *E. vermiformis*-infected mice, αβ T cells play an important role in the memory response to murine coccidiosis since the TCRβ^−/−^ mice were highly susceptible to secondary infection of *E. vermiformis* and remained highly susceptible to subsequent infections, unlike intact mice [38]. Similar to αβ T cells, γδ T cells are involved in memory responses and can rapidly generate and expand IFN-γ production [39].

CMI responses are involved in both the antigen-specific and non-specific activation of T lymphocytes, macrophages, and natural killer (NK) cells [40]. Cytotoxic T lymphocytes and macrophages are specialized in eliminating endogenous and exogenous antigens, respectively [24]. Interest in the gut mucosal lymphoid population, especially IEL NK cells, has increased in studies of human and chicken immunity. Although the exact mechanism has not been established, NK cells, which are mononuclear cells with cytotoxic activity, play a role in *Eimeria* infection through IFN-γ secretion. The observation of subpopulations of NK cells that mediate spontaneous cytotoxicity in chicken intestinal IEL suggests that they are an important component of intestinal immunity [41]. The presence of NK cells in chickens has been demonstrated in the spleen [42], thymus [41], peripheral blood [43], bursa of Fabricius [44], and intestine [45]. NK cell activity in the intestine was higher in the jejunum and ileum than in other parts of the intestine. Lillehoj [46] investigated the role of NK cell activity in *Eimeria*-infected chickens and showed that coccidiosis markedly reduced NK cell activity in splenic lymphocytes and intestinal IEL during the early stages of infection. However, approximately one week after the primary infection, NK cell activity recovered to normal levels, and in the early stages of secondary infection, splenic and intestinal IEL NK cell activity significantly increased. Although local host response depends on the species of *Eimeria* [47], these results support a notion that indicates the important role of NK cells in coccidiosis.

Dendritic cells (DCs) are antigen-presenting cells (APCs) with a unique ability to induce both innate and highly antigen-specific acquired immunity [48] through immune cell proliferation and cytokine production [49]. Because of these abilities, DCs are often called “nature’s adjuvants” [50] and are recognized as an important component of any vaccination strategy. DCs provide an important link between innate and adaptive immunity, and they play a crucial role in the early phase of antigen presentation following coccidiosis as major APCs. APCs contain lysosomal proteases that digest the protein of the captured antigen prior to presentation [51]. In this process, exosomes secreted from APCs activate naive T cells [52]. Based on this mechanism, the utilization of exosomes derived from DCs has been proposed as a novel approach to vaccination against coccidiosis [53]. Chickens immunized with exosomes isolated from chicken intestinal DCs stimulated with a sporozoite mixture of *E. tenella*, *E. maxima*, and *E. acervulina* showed protective immunity compared with non-immunized chickens. The cecal tonsils, Peyer’s patches, and spleens of immunized and *Eimeria*-infected chickens showed an enhanced Th1 immune response. In addition, exosome-immunized chickens showed greater body weight gain, reduced oocyst output, and lower mortality compared with non-immunized chickens. Besides this efficacy against coccidiosis, recent studies have demonstrated that exosomes are useful tools in developing vaccines against other pathogens [54,55]. 

### 3.3. Role of Cytokines and Chemokines 

Various cytokines and chemokines that play a role in coccidiosis infection have been characterized. They include IL-1β, IL-2, IL-4, IL-6, IL-8, IL-10, IL-12, IL-15, 17, and IL-18, IFN-γ, transforming growth factor (TGF)-β1–4, tumor necrosis factor (TNF)-α, TNF-α superfamily 15 (TNFSF15), and lipopolysaccharide-induced TNF-α factor (LITAF) [25,56,57]. These immune molecules are involved in host immunoregulation during primary or secondary infection [56,57,58,59,60,61,62,63,64,65,66]. A recent study investigated the differences in the kinetics of host immune response to coccidiosis in two inbred lines of White Leghorn chickens that exhibit differential resistance (line C.B12) or susceptibility (line 15I) [67]. The RNA-sequencing results suggested that early activation of IFN-γ, IL-10, and immune-related genes, including IL-21, may be the key to resistance to coccidiosis. Increased production of IFN-γ and IL-10 were associated with the resistant line (C.B12) at 2 and 4 days post infection (dpi), and then gradually increased. In contrast, the production of these cytokines was relatively low at 2 and 4 dpi, but dramatically increased at 6 and 8 dpi in the susceptible line (line 15I). These findings indicate that the earlier timing of the critical innate immune response dictates the outcome of coccidiosis infection, and the genetic background of the host is important.

Among the cytokines involved in coccidiosis, IFN-γ is a representative immunomodulator and has received the most attention because of its direct inhibitory effect on the intracellular development of *Eimeria*. In mice, the role of IFN-γ has been well characterized as essential during intestinal parasite infection [68,69]. Gene encoding IFN-γ have been cloned, and their functional role has been investigated in chickens [70]. A considerable expression of IFN-γ expression was detected in the spleen and cecal tonsil but IFN-γ was decreased in the duodenum in the inbred chicken (B2B2) post *E. acervulina* infection [70]. Similar results were observed in *E. tenella*-infected chickens. Upregulated expression of IFN-γ was detected in spleens, cecal tonsils, and in IELs post primary and secondary *E. tenella* infections [40]. Breed et al. [71] demonstrated that IFN-γ is specifically produced by *Eimeria*-stimulated peripheral blood lymphocytes from chickens infected with coccidiosis. Subsequently, IFN-γ was found to be produced by mitogen or antigen-stimulated specific T cells that are present in the blood of chickens infected with *Eimeria* [72]. On the basis of these findings, several studies have attempted to evaluate the potential protective effect of IFN-γ against coccidiosis [34,73]. Chickens treated with recombinant IFN-γ and control chickens were compared after infection with *E. acervulina*. The results demonstrated that chickens treated with recombinant IFN-γ had significantly increased body weight gain compared with that of the control chickens [34]. In addition, IFN-γ inhibited the invasion of sporozoites from *E. tenella* into chicken cells in vitro. [74]. A similar effect was observed in the in vivo experiment. Chickens immunized with recombinant IFN-γ displayed considerably decreased shedding of oocysts and increased body weight compared with non-immunized chickens post *E. acervulina* infection [73].

The structurally homologous proteins IL-1β and IL-18 are notable cytokines involved in the initial inflammatory response. IL-1β induces chemokine production, which promotes the recruitment of inflammatory cells at the inflammation site [75], and IL-18 is involved in IFN-γ secretion [76]. In chickens infected with *E. maxima* and *E. tenella*, highly upregulated expression of IL-1β was found in the duodenum, jejunum, and cecum post primary infection [56,77]. In addition, markedly upregulated expression of IL-1β and IL-18 was detected in *Eimeria*-infected chickens by a chicken macrophage microarray [78]. 

A major cytokine involved in CMI response is IL-2, which is a potent growth factor that stimulates the proliferation of chicken T lymphocytes and the activation of NK cells. Sundick and Gill-Dixon [79] cloned the chicken IL-2 gene and characterized its biological functions [80]. The involvement of IL-2 in poultry coccidiosis has been reported. In chickens infected with *E. acervulina*, increased expression levels of IL-2 were observed after primary and secondary infections [40]. Following primary and secondary coccidiosis, the expression of IL-2 was significantly increased in the duodenum. Notably, in contrast with *E. acervulina* infection, the expression of IL-2 was decreased in chickens infected with *E. tenella*. Whether this difference is attributed to cytokine responses induced by these two different species of *Eimeria* in the different areas of gut or if there are other unknown factors that contribute to different cytokine responses remains to be studied [56]. The importance of IL-2 in host protection against *E. acervulina* infection was demonstrated in a recombinant vaccination experiment where chickens vaccinated with 3-1E recombinant protein showed enhanced protection upon challenge infection when the recombinant protein was given with IL-2 [81].

IL-6 is an important cytokine responsible for the final maturation of B cells that produce antibodies and is mainly produced by endothelial cells, macrophages, and T cells [82]. IL-6 production was observed in sera collected from chickens infected with *E. tenella*, indicating the possibility that IL-6 plays a role in acquired immunity [83]. Moreover, enhanced expression of the IL-6 gene has been reported in the IEL of chickens infected with *E. acervulina*, *E. maxima*, or *E. tenella* [56].

IL-10 is an anti-inflammatory cytokine that downregulates the inflammatory Th1 response by inhibiting the secretion of pro-inflammatory cytokines, such as IL-1β, TNF-α, and IL-6 [84]. In mice, a defect in IL-10 enhances susceptibility to toxoplasma infection [85]. This result suggests that IL-10 downregulates the inflammatory response to reduce host immunopathology in toxoplasmosis infection. In *Eimeria* infection, IL-10 plays a significant role in downregulating harmful inflammatory responses. When comparing two different chicken lines with different coccidiosis susceptibility, susceptible chickens (line 15I) showed greater expression levels of IL-10 in the spleen and gut compared to the resistant chickens (line C.B12) following *E. maxima* infection [62]. Similar results have been reported in *E. tenella*-infected chickens [57]. Compared with the unchallenged chickens, a 20-fold increase in IL-10 expression was observed post primary *E. acervulina* or *E. tenella* infection by qRT-PCR. One possible theory explains the role of IL-10 in coccidiosis infection: *Eimeria* spp. may have evolved to induce IL-10 secretion in the host by stimulating Treg cells to facilitate the invasion of parasites into chicken epithelial cells. Furthermore, IL-10 suppresses the IFN-γ-related Th1 response which plays an important role in protective immunity against intracellular parasitic infection. Morris et al. [86] showed that dietary supplementation with vitamin D reduced production losses by enhancing IL-10 expression and Treg cell activity in *Eimeria*-infected chickens. These results suggest the important roles of Treg cells and IL-10 in the regulation of protective immunity against coccidiosis. 

TNF is primarily produced by activated macrophages but also by other cells such as NK cells, mast cells, and antigen-stimulated T cells [82]. The role of TNF in coccidiosis has been investigated in chickens. The major function of this cytokine is to recruit neutrophils to the site of infection. TNF production was observed in a dose-dependent manner after stimulation with *E. tenella* sporozoites and merozoites in chicken macrophage cells after primary infection. However, TNF production was not observed following secondary infections [87]. An in vivo study was also conducted to examine the role of TNF-like activity in the pathogenesis of coccidiosis in inbred SC chickens. The SC chickens treated with an antibody against TNF showed less body weight loss during *E. tenella* infection, indicating the involvement of TNF in coccidiosis pathogenesis [88].

Chemokines are important mediators that induce host defense mechanisms by facilitating the migration of leukocytes to inflammation sites [89]. These proteins are commonly produced by various cell types in response to endogenous and exogenous mediators such as IL-1, IFN-γ, TNF, and the platelet-derived growth factor [90], and approximately 23 chemokines have been identified in chickens [91]. C and CC chemokines are involved in regulating T lymphocytes, monocytes, eosinophils, and basophils [92], and CXC chemokines regulate neutrophil migration [72]. In vitro, chemokine production was observed in macrophages stimulated by *Eimeria* sporozoites [78]. In addition, upregulated expression of mRNAs encoding MIP-1b and K203 genes was observed in the ceca of chickens infected with *E. tenella* and in the jejunum following *E. maxima* infection [77].

### 3.4. Role of the Gut Microbiome in Host Response 

The gut microbiota is a complex ecosystem that influences the physiological response of the host, including their immune development and function, nutrition and metabolism, and pathogen exclusion [93]. One of the main functions of the gut microbiota is to prevent the dominance and colonization of pathogenic bacteria by maintaining intestinal homeostasis through the competitive exclusion of pathogenic microbes [94]. Several studies have demonstrated that differences in energy harvesting by the gut microbiota can affect energy balance, growth performance, and feed efficiency in chickens [95,96,97]. Advances in next-generation sequencing led to the identification of gastrointestinal tract-associated microorganisms and their potential influence on human and animal health [98,99]. Using 16S rRNA gene sequencing, Huang et al. (2018) investigated the change in the overall intestinal microbiome in chickens infected with *E. tenella* and found significant dysbiosis in cecal microbiota. *E. tenella* infection reduced the levels of nonpathogenic bacteria including *Lactobacillus* and *Faecalibacterium* and increased the levels of pathogenic bacteria such as *Clostridium, Lysinibacillus*, and *Escherichia*. Similar changes in fecal microbiota were observed in several commercial chicken spp. (Cobb500, Arbor Acres broilers, and White Leghorn chickens) indicating that dysbiosis in the gut was directly induced by *E. tenella* infection [100,101]. Microbiota clustered in the cecum or colon, such as *Ruminococcaceae*, function to generate energy and nutrients by decomposing non-starch polysaccharides into simple sugars [102]. *Faecalibacterium* aids in the fermentation of these sugars and produces butyrate and essential amino acids, which play an important role in relieving chronic inflammation and reducing damage from *E. tenella* infection [103]. 

In a necrotic enteritis (NE) disease model using the coinfection of *E. maxima* and *Clostridium perfringens*, increasing *Clostridium sensu stricto 1*, *Escherichia Shigella*, and *Weissella* populations and decreasing *Lactobacillus* populations were seen in the jejunum [104]. The reduction of commensal bacteria, such as *Lactobacillus* spp., affects microbial diversity and disrupts significant metabolic processes that provide energy and sources or carbon [97]. In addition, the reduction of *Lactobacillus reuteri*, which produces an antimicrobial substance called reuteri, can lead to the proliferation of other bacterial species, resulting in the loss of host defense capabilities [105]. For example, an in vitro study reported that *Lactobacillus* spp. significantly inhibited the invasion of *E. tenella* sporozoites in Madin-Darby bovine kidney cells [106]. Cui et al. [107] investigated the effect of *E. tenella* infection on cecal microbiota in specific-pathogen-free chickens and observed a reduction in potentially beneficial bacteria (i.e., *Ruminococcaceae, Anaeroplasma, Phascolarctobacterium, Faecalibacterium, Coprococcus Ruminococcus*, and *Blautia*). These bacteria inhibit the proliferation of conditional pathogenic bacteria through substances with broad-spectrum antimicrobial activity and reduce the production of harmful substances, such as endotoxins [108]. In contrast with a reduction in potentially beneficial bacteria, many abundant conditional pathogenic bacteria, such as *Escherichia−Shigella, Enterococcus, Bacillus*, and *Staphylococcus*, have been detected in the cecum of *E. tenella*-infected chicks [103,107]. 

Dysbiosis associated with coccidiosis increases host susceptibility to other pathogens. Infection with *Eimeria* parasites compromises intestinal integrity and affects nutrient absorption by reducing the function of the intestinal barrier and leads to a bacterial imbalance affecting bacterial-dependent metabolic processes in the gastrointestinal tract. Consequently, an intestinal bacterial imbalance increases the risk of susceptibility to other diseases by disrupting the gut homeostasis of the host [109]. Due to the detrimental outcome of severe coccidiosis on commercial poultry production, further studies are needed to understand both the intricate interactions between *Eimeria* parasites and the gut microbiota and the effects of coccidiosis on host physiological responses, including nutrient absorption and local immunity.

## 4. Recombinant Vaccine Antigens of *Eimeria*

### 4.1. Candidate Antigens for Vaccine Development

Advancements in molecular biology, genetics, biochemistry, and genetic engineering technology have revolutionized research and vaccine development in the animal vaccine industry, and attention is focused on developing a safe, efficient vaccine with a low risk of side effects. Coccidiosis is a common intestinal disease of poultry that causes significant economic losses in the global poultry industry. Coccidiosis has been traditionally controlled by live and attenuated *Eimeria* strains or anticoccidial drugs such as diclazuril, toltrazuril, and ionophores [110,111]. Live and attenuated parasite vaccines are highly effective for controlling field coccidiosis, but the high cost of vaccine production and their limited availability, not to mention the emergence of drug-resistant *Eimeria* strains, have generated increased interest in recombinant vaccines as an alternative strategy to control coccidiosis [112]. Numerous *Eimeria* antigens have been identified as effective anticoccidial vaccine candidates, but there are not yet commercially available recombinant vaccines in the market [113]. Most of the *Eimeria* proteins identified as anticoccidial agents target surface and internal parasite antigens (Table 1). Due to the fact that they are naturally exposed to the host’s immune system during *Eimeria* invasion and reproduction, these are suitable targets that can trigger the host’s protective immune response.

Liu et al. [114] identified five *Eimeria* immunodominant antigens, including elongation factor 2 (EF-2), 14-3-3 protein, ubiquitin-conjugating enzyme domain-containing protein (UCE), and glyceraldehyde-3-phosphate dehydrogenase (GAPDH), by comparing the amino acid sequences of three *Eimeria* spp. (*E. acervulina*, *E. maxima*, and *E. tenella*). Lin et al. [115] cloned the EF-1α gene from sporozoites of *E. tenella* and evaluated its protective effect in chickens infected with *E. maxima* and *E. tenella*. Chickens immunized with recombinant EF-1α exhibited greater body weight gain, improved serum antibody production against EF-1α, and decreased fecal oocyst shedding post *Eimeria* challenge with either *E. maxima* or *E. tenella* compared to the unimmunized chickens.

Tian et al. [116] evaluated the immunogenicity and protective efficacy of the GAPDH gene cloned from *E. acervulina* and *E. maxima* in chickens infected with *E. tenella*, *E. acervulina*, *E. maxima*, or a mix of these. Chickens vaccinated with recombinant GAPDH showed an enhanced proportion of CD4^+^ and CD8^+^ T lymphocytes, increased cytokine secretion (IFN-γ, IL-2, IL-4), and a greater IgG antibody production compared to the non-immunized chickens. In addition, vaccination increased weight gain, decreased fecal oocyst shedding, and mitigated gut lesions compared to the non-immunized chickens. 

The highly immunogenic protein SO7 is a refractory body protein that contains an epitope commonly shared among all *Eimeria* spp. that infect domestic fowl [117]. Chickens which were immunized with SO7 showed reduced parasite excretion compared to the non-immunized control chickens [118]. 

In another in vivo trial, chickens vaccinated with recombinant SO7 protein showed a significant reduction in oocyst output and increased weight gain compared with non-vaccinated chickens [119]. In addition, increased serum IgY and IFN-γ levels and lymphocyte proliferation were associated with the SO7-immunized chickens.

Two gametocyte antigens (GAM56 and GAM82) from the sexual stages of *Eimeria* parasites are important components of the oocyst wall and potential vaccine candidates as transmission-blocking vaccines against coccidiosis [120,121]. Chickens which were vaccinated with these two antigens showed significantly decreased oocyst shedding and enhanced serum antibody and lymphocyte proliferation response in *E. maxima*-infected chickens [120,122,123]. In addition, transgenic plants (tobacco leaves) expressing Gam 56 and Gam 82 antigens elicited a protective immune response in immunized chickens (but not in non-vaccinated chickens) as measured by increased body weight gain and reduced fecal oocyst output [124]. Liu et al. [125] cloned and expressed a gametocyte gene from *E. necatrix* (Engam22) which showed 97.7% identity to that of Etgam22 of *E. tenella* and this antigen was recognized in serum from chickens immunized with *E. necatrix*, *E. tenella*, and *E. maxima*. Furthermore, the Etgam22 antigen of *E. tenella* was an ortholog of *E. necatrix* gametocytes, indicating a potential target for future recombinant subunit vaccines against coccidiosis. The immunoprophylactic efficacy of recombinant gametocyte antigen 22 from *E. tenella* (EtGam22) was evaluated in chickens following *E. tenella* infection [126]. Vaccination with EtGam22 induced strong cytokine production, including IL-2, IL-4, TGF-β, and IFN-γ, and showed higher peripheral blood lymphocyte proliferation. A significant reduction in oocyst output and a lower weight loss than in the non-immunized challenged control was observed. Similarly, Zhao et al. [127] developed recombinant protein and DNA vaccines using *E. tenella* surface antigens 4 (EtSAG4) and showed that the EtSAG4 recombinant protein induced a significant (*p* < 0.05) protective immunity based on IgY and IFN-γ production, body weight gain, and reduced oocyst output in *E. tenella*-infected Cobb broilers. However, the DNA vaccine (pEGFP-N1-EtSAG4 plasmids) induced much higher levels of protection against *E. tenella* than the EtSAG4 recombinant antigen vaccination.

Profilin, also called 3-1E, is one of the most widely evaluated subunit vaccine candidates [93,113,128]. 3-1E is a surface antigen synthesized in all stages of *E. tenella* and was found in both merozoites and sporozoites of *E. acervulina* and *E. maxima* [129]. The 3-1E protein contains a putative conserved domain for actin-regulatory protein profilin [81] and induces cell-mediated immunity [130]. In several studies, chickens intramuscularly immunized with the recombinant 3-1E antigen have shown greater body weight gain and lower fecal oocyst shedding post *Eimeria* challenge. In addition, improved serum antibody levels of IgG and higher cytokine production than in non-immunized chickens were observed [93,130,131]. Employing in ovo delivery, Ding and colleagues (2004) evaluated the recombinant 3-1E protein from *E. acervulina* against coccidiosis in combination with plasmid carrying a gene encoding IL-1, IL-2, IL-6, IL-8, IL-15, IL-16, IL-17, IL-18, or IFN-γ. The outcome of the study demonstrated that the 3-1E in ovo vaccination elicited protective immunity against *E. acervulina* infection as measured by decreased fecal oocyst output and a lower body weight loss than in non-immunized chickens. Moreover, the co-injection of the 3-1E vaccine with a plasmid expressing a chicken cytokine such as IL-2, IL-15, IL-17, IL-18, or IFN-γ further enhanced vaccine efficacy [81]. In a recent in vivo trial, Lillehoj et al. [132] demonstrated that recombinant 3-1E vaccine together with the recombinant necrotic enteritis B-like toxin protein induced significant protection against necrotic enteritis challenge infection. 

The protective effects of α-tubulin protein from *E. acervulina* were demonstrated by Ding et al. [133]. Chickens immunized with recombinant α-tubulin protein expressed in *E. coli* (BL21) showed a 36% reduction in fecal oocyst shedding, decreased intestinal lesion scores, and increased body weight gains in comparison with the non-immunized chickens. Subsequent studies have demonstrated that defined antigens, such as apical membrane antigen 1 (AMA1) and immune mapped Protein-1 (IMP1) from *E. maxima*, are potential vaccine candidates [112,134,135]. Chickens immunized with the AMA1 antigen showed strong humoral and cellular responses against *E. maxima* infection [134], whereas partial protection against high level *E. maxima* challenge was observed following vaccination with transgenic *E. tenella* oocysts expressing the AMA1 and IMP1 antigens of *E. maxima* in a broiler chicken model of coccidiosis [112].

In comparison to using live and attenuated parasite vaccines, recombinant antigens are safe and more immunogenic since they are selected on the basis of their ability to induce various aspects of protective host immunity. However, since recombinant antigens do not elicit a wide spectrum of protective immune responses as living or attenuated parasite vaccines, there are some limitations associated with using a single recombinant *Eimeria* protein to protect against coccidiosis. Furthermore, variations in *Eimeria* strains as well as the high cost of producing multiple strains of *Eimeria* parasites will be limiting factors. Therefore, novel strategies to improve the efficacy of recombinant vaccination need to be developed in order for recombinant vaccine approaches to be successful in the field when it comes to vaccinating against coccidiosis [81].

### 4.2. Molecular Vaccines against Coccidiosis

The principle of a DNA vaccine is to use a plasmid vector to transfer genes, since DNA must enter the cell in order to express the antigen it carries [136]. Lillehoj et al. (2000) showed that the subcutaneous immunization of young chickens with a cDNA encoding 3-1E *E. acervulina* protein elicited significant protective immunity against *E. acervulina* challenge infection. The 3-1E cDNA vaccine significantly reduced fecal oocyst shedding, and the efficacy of the vaccine was enhanced when the 3-1E cDNA vaccine was co-administered with an IFN-γ- or IL-2-expressing plasmid [136]. Embryo vaccination using 3-1E and cytokine-encoding plasmids (IL-1, IL-2, IL-15, or IFN-γ genes) against *E. acervulina* infection showed an improved serum antibody response and higher levels of protection based on growth performance and parasite fecundity [137]. In another study, a combination of two DNA vaccines encoding TA4 and Et1A sporozoites of *E. tenella* also induced protective immunity against coccidiosis based on reduced oocyst shedding and improved growth performance following *E. tenella* infection [138]. The potential vaccine efficacy of the 14-3-3 protein of *E. maxima* which is involved in apoptotic cell death, cell cycle control, and mitogenic signal transduction was evaluated [139]. The immunization of young chickens with the Em14-3-3 gene from *E. maxima* in the pVAX1 vector reduced jejunum lesions and body weight loss following challenge infection with *E. maxima*. Furthermore, chickens vaccinated with Em14-3-3 showed higher percentages of circulating CD4^+^ lymphocytes with enhanced IFN-γ and TGF-β production compared to control chickens immunized with PBS alone. Zhang et al. [140] investigated various optimization strategies using DNA immunization against coccidiosis. Recombinant vector pVAX1-pEtK2-IL-2, which was constructed by cloning the pEtK2 antigen gene of *E. tenella* and chicken IL-2 gene in pVAX1, elicited a significant level of protection against *E. tenella* infection, as indicated by a higher survival rate, greater average body weight gain, and a lower gut lesion score in vaccinated chickens. In addition, intramuscular delivery was the most efficient route for inducing a protective immune response, and two injections with 80 µg of each DNA were highly effective in terms of protection against coccidiosis challenge infection. Chickens vaccinated with a DNA vaccine of *E. tenella* surface antigen 4 (EtSAG4) exhibited higher levels of secretory IgY antibodies, a greater IL-17 and IFN-γ cytokine response, and better clinical performance in terms of body weight, oocyst output, and gut lesion scores compared to the non-immunized control [127]. 

### 4.3. Adjuvants, Cytokines and the Modes of Recombinant Vaccine Immunization

Although many recombinant vaccines against coccidiosis have been reported, their effectiveness has not been comparable to live and attenuated vaccines in terms of the level of protection. Therefore, there is a timely need to evaluate various adjuvants and antigen delivery systems to maximize the efficacy of recombinant vaccines [20]. Min et al. [128] evaluated the adjuvant effects of various cytokines using plasmid genes, including IL-1β, IL-2, IL-8, IL-15, IFN (α/γ), TGF-β4, and lymphotactin to improve the efficacy of the 3-1E cDNA *Eimeria* vaccine. Using a novel adjuvant delivery system, QCDC, Lee et al. [141] demonstrated an enhanced protective effect as measured by body weight and intestinal cytokine response following embryo vaccination with a profilin antigen from *Eimeria*. In other vaccine trials, Montanide IMS or an ISA series of adjuvants that comprise a mixture of a defined, enriched light mineral oil enhanced the immunogenicity of recombinant vaccines and elicited desired immune responses [8,119,122,132]. Interestingly, Zhang et al. [142] evaluated the efficacy of profilin recombinant protein with a phytochemical adjuvant, namely ginsenosides extracted from the root of a ginseng plant, against *E. tenella* infection. Chickens co-immunized with profilin and ginsenoside extract showed greater antibody production, reduced gut lesions, and lowered oocyst fecundity compared to the chickens immunized with profilin only. 

### 4.4. Delivery Vectors for Recombinant Vaccines

The efficacy of recombinant vaccines can be improved by optimizing the antigen presentation system and using an antigen delivery system that induces a broader protective immune response [143]. Various types of delivery vectors including eukaryotic expression vectors (*Salmonella* strains and pVAX1), a yeast vector (*Saccharomyces cerevisiae*), or bacterial vectors (pMV361 and pET32a) were evaluated in combination with immunogenic *Eimeria* antigens [114,144,145]. Immunization with EtMic2 microneme protein delivered in a *Saccharomyces cerevisiae* vector induced a higher level of protection with increased weight gain and reduced cecal pathology and fecal oocyst shedding compared with non-immunized chickens [144]. Furthermore, *E. maxima* 14-3-3 antigen in pVAX1 and pET32a (+) delivery vectors induced significant protective response in *E. maxima*-infected chickens, including reduced oocyst production, lower jejunum lesions, and lower body weight loss, and better immune responses as indicated by higher percentages of CD4^+^ cells and enhanced serum antibody titers [114].

Attenuated strains of *Salmonella enterica serovar* Typhi (S. Typhi) or S. *Typhimurium*, have been successfully used to deliver heterologous antigens from various pathogens [146] to stimulate protective mucosal immune response [129]. Shivaramaiah et al. [147] used *Salmonella enteritidis* (*S. enteritidis*) to deliver *E. maxima* TRAP family protein (EmTFP250) to demonstrate its vaccine efficacy against coccidiosis as measured by improved weight gain and reduced mortality. Wang et al. [145] used the pMV361 vector to produce rBCG pMV361-rho and pMV361-rho-IL2 vaccines and demonstrated their ability to generate improved protective immunity against *E. tenella* infection as measured by reduced cecal lesions and oocyst output.

The possibility of using *Eimeria* parasites as vectors carrying other antigens has also been explored [114,130,148]. There are many advantages associated with using the *Eimeria* parasite delivery system due to its strict host specificity, safety, and large genome size [149,150]. Tang et al. [151] and Pastor-Fernández et al. [112] demonstrated the feasibility of using *E. tenella* oocysts as vaccine vectors to express the *E. maxima* antigens EmAMA1 and EmIMP1. The oral immunization of chickens with *E. tenella* vector carrying a *Campylobacter* jejuni antigen, CjaA, induced approximately 90% immune protection against *Campylobacter* jejuni infection compared with non-immunized or wild-type *E. tenella*-immunized chickens [148]. In another report, transgenic *E. tenella* oocysts expressing two different viral antigens from infectious bursal disease virus (IBDV) and infectious laryngotracheitis virus (ILTV) elicited a serum antibody response [152].

Studies utilizing plant-derived vectors have also been reported [124]. Plant-based vaccine technology provides high replication ability, cost-effectiveness, and are free from contamination by animal pathogens [153]. Kota et al. [124] evaluated the efficacy of *E. maxima* gametocyte antigen (Gam82) which was expressed in tobacco leaves and showed its protective effects in terms of body weight gain and parasite fecundity.

## 5. Alternatives to Antibiotic (ATA) Strategies to Control Coccidiosis

**Figure 1:**
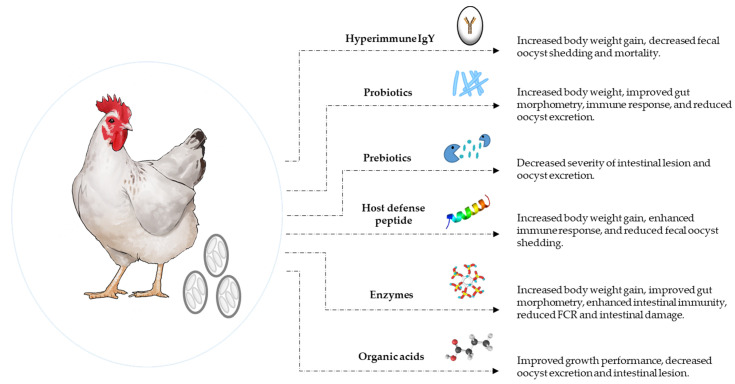
Antibiotic alternatives to control coccidiosis in chickens. Various novel strategies including hyperimmune IgY [166,167,168], probiotics [170,171,173,174,175], prebiotics [181,182,183], host defense peptides [187,188,189], enzymes [180,181,182,183,184,185,186,187,188,189,190,191,192,193,194,195,196,197,198,199,200], and organic acids [192] have been developed for coccidiosis control in chickens.

In addition to vaccine development, various antibiotic alternative strategies, including hyperimmune egg yolk antibodies, phytochemicals, probiotics, prebiotics, and host defense peptides, have been successfully developed to mitigate coccidiosis [154]. In addition, the utilization of various organic acids and feed enzymes has attracted considerable attention (Figure 1). Alternatives to antibiotics are broadly defined as any substance that can serve as a substitute for therapeutic drugs, which are increasingly becoming ineffective against pathogenic bacteria, viruses, or parasites [155]. A plethora of studies has been conducted with different feed additives to evaluate their potential as antibiotic alternative strategies against coccidiosis in poultry. 

Most of these have high potential because they reduce the pathogenic load, mitigate gut damage, and enhance local immunity in the intestines of poultry [154]. In addition, the combination of different antibiotic alternative strategies, such as prebiotics and probiotics, show a significant synergistic effect [156,157]. However, careful attention is required when selecting combinations of various antibiotic alternatives since we need to understand their mechanisms of action and evaluate their effectiveness for various field conditions through sufficient research in target animals.

### 5.1. Hyperimmune Egg Yolk Antibodies

Passive immunization with pathogen-specific IgY antibodies is an effective prevention and treatment strategy which is effective as a potential antibiotic alternative for the treatment and prevention of various human and animal diseases [158]. IgY antibodies, considered as the functional equivalent of mammalian IgG, have been successfully employed in the prophylaxis and treatment of various enteric infections in swine and cattle [159,160]. In addition, the successful application of IgY in disease treatment and the prevention of enteric pathogen infections in poultry and humans has already been achieved [161,162]. 

Passive immunization utilizing pathogen-specific egg yolk antibodies (IgY) is attracting increasing interest as an alternative strategy to AGPs to improve growth and feed efficiency in poultry [163,164,165]. Since maternal IgY is concentrated in the yolk sac during embryonic development, it can be easily extracted and purified. In addition, antibodies are produced by a relatively non-invasive method, for which large-scale production is also possible, offering a practical alternative for antibody production [158]. As IgY production technology advances, this technology can synergize with vaccines to improve the animal’s capability to protect against various infections where antibodies play a role. [156,157].

Lee et al. [166] reported that diet supplementation with 10% or 20% hyperimmune IgY egg yolk powder (Supracox^®^) protected chickens from a subsequent challenge of *E. acervulina*. Chickens fed diets supplemented with 10% or 20% Supracox^®^ showed greater weight gain and lower oocyst production than chickens fed the basal diet. For chickens fed lower levels of Supracox^®^ (0.01, 0.02, 0.05, or 0.5%), fecal oocyst shedding was markedly reduced, but no effect on body weight gain in *E. acervulina* infection was observed. A similar effect was observed in *E. maxima*- and *E. tenella*-infected chickens with less body weight loss, alleviated intestinal lesions, and fecal oocyst production when compared to chickens fed a basal diet [167]. In both studies, results were obtained via hyperimmune IgY produced from hens immunized by three *Eimeria* spp. (*E. acervulina*, *E. maxima*, and *E. tenella*). In another study, Xu et al. [168] utilized hyperimmune IgY produced from hens immunized with five *Eimeria* spp. (*E. acervulina*, *E. maxima*, *E. tenella*, *E. necatrix*, and *E. praecox*) and observed its protective effect in *E. tenella*-infected chickens. Chickens fed diets supplemented with hyperimmune IgY showed greater body weight gain, reduced mortality, mitigated cecal lesion scores, and lower oocyst output compared with chickens fed a basal diet. The use of IgY antibodies in passive immunization offers several advantages in that it is environmentally friendly, nontoxic, and reduces the numbers of animals required for antibody production. The successful application of IgY in disease treatment and prevention of enteric pathogen infections in poultry and humans has already been achieved, but a novel delivery system will enhance its field application.

### 5.2. Probiotics

Probiotics are live microbial feed supplements which beneficially affect the host animal by improving its intestinal microbial balance. Prebiotics, by contrast, are non-digestible feed ingredients that beneficially affect the host by selectively stimulating the growth and/or activity of one or a limited number of bacteria in the gut [169,170,171]. Synergetic combinations of probiotics and prebiotics are known as synbiotics. Probiotics, prebiotics, and synbiotics reportedly reduce the numbers of pathogenic microorganisms in the gastrointestinal tract while simultaneously enhancing the beneficial microbial flora leading to improved growth performance without the disease. 

In humans and animals, gut microflora is an important component of a primary line of defense. In poultry, probiotic supplementation of the intestinal microflora has been shown to positively regulate the microbiota in the intestine and inhibit the proliferation of pathogen colonies [154]. Therefore, the aim of the use and development of probiotics is to develop and maintain beneficial intestinal microflora, improving host resistance to intestinal pathogens [170,171,172]. Although numerous studies have demonstrated disease prevention and boosted immune systems resulting from the oral administration of probiotics, few studies have investigated their beneficial effects against coccidiosis [170,171]. 

Dalloul et al. [173,174] conducted several studies to show the beneficial effects of *Lactobacillus*-based probiotics in stimulating local immunity against coccidiosis. Lee et al. [171] reported that dietary *bacillus*-based direct-fed microbials (Bs2084, LSSAO1, 3AP4, Bs18, 15AP4, 22CP1, Bs27, and Bs278) reduced the clinical signs and increased immunity in *E. maxima*-challenged chickens. In a study using a commercial product, *Pediococcus*, and *Saccharomyces*-based probiotics (MitoMax^®^), 1.0% or 0.1% MitoMax-supplemented diets decreased oocyst shedding in *E. acervuline*- or *E. tenella*-infected chickens. In addition, a 0.1% MitoMax-supplemented diet increased the serum levels of *Eimeria*-specific antibodies [170]. In a recent study, the effects of dietary *Bacillus subtilis* 1781 or 747 supplementation on *E. maxima*-infected chickens was investigated [175]. Chickens fed a diet supplemented with *B. subtilis* 1781 or 747 showed improved growth performance to a level comparable to that induced by antibiotics (virginiamycin or BMD) without *E. maxima* infection. Post *E. maxima* infection, diet supplementation with *B. subtilis* 747 enhanced intestinal immunity and epithelial barrier integrity. With the development of “omics” technologies related to the investigation of gut health, the term “postbiotics” was coined and defined as a novel class of feed additives that are generally produced by beneficial gut microbes and which exert a positive influence on host health. In a recent in vivo feeding trial, the dietary feeding of *Bacillus subtilis* 1781 strain induced an alteration of chicken gut metabolites which was associated with the growth- and immune-promoting effects of *B. subtilis* probiotics [169]. Interestingly, among the highly altered gut metabolites, maltol was one of the significantly increased metabolites that mediated various physiological functions associated with antioxidant and anti-inflammatory activities. These studies suggest that further investigation of the small molecular weight gut metabolites associated with the beneficial effects of probiotics will lead to the identification of novel antibiotic alternative postbiotics that can be used to improve the growth and immunity of chickens. 

### 5.3. Prebiotics

Prebiotics are non-digestible oligosaccharides and potential modulators of intestinal microflora that promote the growth of probiotics and their activities in the gut [176]. Arabinoxylooligosaccharides (AOS), fructooligosaccharides (FOS), isomaltooligosaccharides (IMOS), mannan-oligosaccharides (MOS), soy oligosaccharides (SOS), xylooligosaccharides (XOS), pyrodextrins, and inulin are prebiotics commonly used in poultry [177,178]. Most of these prebiotics are derived from plants such as artichoke, onion, garlic, chicory, leek, and tomatoes [177], and some studies have reported that the dietary supplementation of chicken feed with these prebiotics has improved the host defense system against pathogen infection and decreased the mortality rate [179,180]. Prebiotics selectively stimulate the growth of beneficial bacteria in the intestinal system of the gut and increase the number of beneficial microbiota. Consequently, harmful pathogens are excluded because of the dominance of beneficial microbiota in the intestinal tract of chickens [178].

In some studies, the inhibitory effects of prebiotics against *Eimeria* infection in poultry have been reported [181,182,183]. In an experiment, dietary MOS (10 g/kg feed) reduced oocyst fecal shedding and diminished the severity of *E. acervulina* lesions in chickens orally infected with three *Eimeria* spp. mixtures (*E. acervulina*, *E. maxima*, and *E. tenella*) at subclinical doses (900, 570, and 170, respectively) [183]. Bozkurt et al. [182] reported that the dietary supplementation of MOS (1 g/kg feed) positively influenced growth and feed conversion efficacy and reduced the severity of lesions in mixed *Eimeria* spp. infection. In a subsequent study, the efficacy of an in ovo-delivered commercial prebiotic, Bi2tos (trans-galactooligosaccharides), was examined [181]. In in ovo administration, prebiotics reduced the severity of intestinal lesions and oocyst excretion induced by three different spp. of *Eimeria*. Prebiotics and probiotics have many antibacterial mechanisms in common. Accordingly, the use of prebiotics is emerging as a new approach to control coccidiosis.

### 5.4. Host Defense Peptides

Host defense peptides (HDPs) are important effector molecules of the innate immune system; they are present in several organisms and play a significant role in the first line of host defense [184]. They are also called antimicrobial peptides because of their ability to inhibit bacterial growth in vitro, and four major structural groups—amphipathic α-helical, β-sheet, β-hairpin or loop, and extended variants—have been found [185]. HDP peptides possess broad-spectrum antibiotic activity against enveloped viruses, fungi, gram-positive and gram-negative bacteria, and mycobacteria [186]. The involvement of HDPs against coccidiosis in chickens has been observed in a small number of studies [187,188,189]. 

Antimicrobial peptides (AMPs) are another effective alternative to antibiotics since they have broad spectrum of bactericidal activity and selectivity. Cationic antimicrobial peptides are highly conserved in all organisms and are effective against many bacteria, including multidrug-resistant strains. AMPs act by disrupting the bacterial membrane based on their cationic nature [190]. The immunomodulatory activity of cationic AMPs is complex and includes anti-infective immune modulation, such as the induction of chemokines and cytokines, pro/anti-inflammatory activity, direct chemotaxis, wound healing, angiogenesis, apoptotic activity, and adjuvant activity. In our early studies, we found that chicken NK-lysin (cNK-lysin), a cationic amphiphilic AMP and homologue of human granulysin, exhibits cytolytic activities against tumor cells and *Eimeria* parasites. Chicken NK-lysin-2 (cNK-2) is a natural lytic peptide that has been reported to have effective cytotoxicity against apicomplexan parasites such as *Eimeria*, by disrupting the sporozoite membrane [190]. cNK-2, derived from the cationic core region of the cNK-lysin protein and secreted from chicken cytotoxic lymphocytes during coccidiosis, has been shown to successfully destroy *Eimeria* spp. both in vitro and in vivo [185]. Hong et al. [187] reported that the expression of NK-lysin, an antimicrobial and anti-tumor protein expressed by NK cells and T lymphocytes, was upregulated by more than three-fold in CD4^+^ and CD8^+^ intestinal IELs post infection with *E. acervulina*, *E. maxima*, and *E. tenella*. Subsequently, Lee et al. [188] investigated the in vitro and in vivo antiparasitic capability of a synthetic peptide (cNK-2) including a predicted membrane-permeating, amphipathic alpha-helix of the full-length chicken NK-lysin. In vitro treatment with the cNK-2 peptide showed dose- and time-dependent cytotoxic activity against sporozoites of *E. tenella* and *E. acervulina*. In addition, the disruption of the outer plasma membrane and loss of intracellular contents of *E. tenella* sporozoites were detected by transmission electron microscopy. The in vivo administration of the cNK-2 peptide demonstrated a novel anti-parasiticidal activity, measured by increased body weight gain and reduced fecal oocyst shedding. 

In a sustainable delivery system, AMPs not only promote host local immunity but also reduce parasite growth and promote a healthy gut microbial community. Moreover, effective industry-friendly delivery strategies for antibiotic alternatives will reduce the cost of labor and increase the effectiveness of antibiotic-free animal production. Recently, an oral delivery strategy using *Bacillus* spores to the intestine where *Eimeria* parasites interact with the host’s gut epithelial cells has been reported [5]. A stable strain of *B. subtilis* that carries the cNK-2 peptide was orally given to young broiler chickens, following which they were challenged with viable *E. acervulina* oocysts. The results showed that *B. subtilis*-cNK-2 treatment is a promising and effective alternative strategy to replace antibiotics against coccidiosis based on its ability to reduce parasite survival, reduce coccidiosis-induced body weight loss, and decrease gut damage based on the enhanced expression of proteins associated with gut integrity and intestinal health. Su et al. [189] profiled the expression of two HDPs, avian beta defensin (AvBD) and liver expressed antibacterial peptide 2 (LEAP2), which are part of the innate immune system post *Eimeria* spp. challenge. Overall, the AvBD response to an *Eimeria* challenge was inconsistent, whereas the expression of LEAP2 was continuously downregulated in the duodenum, jejunum, ileum, and ceca of chickens infected with *E. acervulina*, *E. maxima*, or *E. tenella*. This result suggests that LEAP2 is closely involved in the control of *Eimeria* infection. 

### 5.5. Organic Acids and other Feed Additives

The use of organic acids has resulted in considerable achievements in poultry and swine production. Dietary supplementation of acetic acid in broiler chickens was shown to improve growth performance (weight gain and feed consumption ratio) and pathological parameters (mortality, lesion scores, and oocyst shedding) [191]. Butyric acid glycerides (lactobutyrin) and clopidol showed potential anticoccidial effects in *E. maxima*-infected chickens, measured by lower oocyst shedding and the mitigation of lesions [192]. The mechanism of action of organic acids is not clearly understood. However, organic acids improve performance and disease resistance in poultry by reducing the pH level of the gastrointestinal tract and changing the composition of the gut microbiome [193]. However, higher concentrations of organic acids can have a negative impact on growth performance [194]. No organic acids are currently commercially available for the treatment of coccidiosis, and most experimental results have been verified from intensively farmed broilers.

Coccidiosis infection noticeably increases the maintenance energy requirement of the host [195]. In addition, infections with *E. acervulina* and *E. maxima* were found to decrease the transcript levels of digestive enzymes and nutrient transporters [196,197]. A few studies have investigated the utility of feed enzymes as a strategic alternative to complement deficient enzymes due to coccidiosis infection. [104,198]. Dietary supplementation with β-mannanase in chickens challenged with commercial live-attenuated coccidiosis vaccine (20x) increased the proportions of beneficial intestinal groups, such as *Lactobacillus*, *Ruminococcaceae*, and *Akkermansia*, and decreased *Bacteroides*, which is responsible for poor feed efficiency in chickens [104]. Furthermore, although it did not affect the productive performance of chickens, supplementation with β-mannanase improved intestinal health, measured by improved villi, namely the crypt proportion and increased number of goblet cells in the intestinal mucosa [198]. Dersjant-Li et al. indicated that the damage and performance losses induced by coccidiosis could be reduced by using enzymes in combination with *Bacillus* spp. In chickens infected with a six-fold concentration of coccidial vaccine, an enzyme blend (xylanase, amylase, and protease) in combination with three strains of *Bacillus* spp. reduced the inflammatory response and maintained a performance similar to that of unchallenged chickens [199].

Beyond the above-mentioned feed additives, new technologies are being applied to identify novel feed additives that trigger natural, physiological mechanisms in animals to cope with enteric infections, including quorum sensing, bacteriophages, and small molecular weights chemicals.

Antibiotic alternatives represent a major unmet need for the livestock sector. However, the factors predicting their success or failure are complex. A framework for the evaluation of alternative candidates that may empower federal agencies, philanthropic organizations, and other key stakeholders to prioritize investments in antibiotic alternatives consistently and transparently was proposed in a recent workshop [200]. This framework first considers the overall costs and benefits related to the new alternative, because economic viability is the foundational for ultimate commercial success and this information may be readily available prior to or during early research and development. Ultimately, bringing an ATA to the market is an extremely complex process, involving the evaluation of product safety, efficacy, acceptability, and practicality. Therefore, the potential success of a new alternative may be best evaluated from multiple perspectives, an approach that we replicated in our original survey and workshop design and encourage in the evaluation of alternatives [200]. Research funders may, for instance, start to involve farmers, veterinarians, and farm advisors more closely in early funding decisions. Developing new antibiotic alternatives is a challenging issue but holds considerable promise for animal health and the fight to combat antibiotic resistance. This framework will empower research funders to evaluate alternatives during early research and development and to dedicate scarce funding to the most promising ATAs.

## 6. Concluding Remarks

Chicken is one of the most commonly available meat protein sources, and its demand is steadily increasing globally. The decline in the use of AGPs and the lack of discovery of new antimicrobials provide impetus to find alternatives to antibiotics. The recombinant vaccine strategy is one of the logical antibiotic-independent alternatives that has engendered much interest from the pharmaceutical industry ever since the technological advancement in genetic engineering was achieved. However, it has been difficult to find the nature of immunogenic parasite antigens, that elicit a wide spectrum of host protective immune responses, which is comparable to those induced by live parasites. Furthermore, the high-level production of these recombinant proteins is cost-prohibitive and there is a lack of appropriate delivery systems for recombinant proteins to ensure sustainable large flock field vaccination due to the high levels of mutability of *Eimeria* parasites. Due to the complexity of host-parasite immunobiology, developing novel strategies for the effective prevention of coccidiosis will require a systematic, detailed analysis of host–parasite interactions at the molecular and cellular levels. Furthermore, a better understanding of the role of gut microbiota in the development and functioning of GALT, the interaction of the gut immune system with gut microbiota, and new concepts on gut–brain interaction is needed. Using optimal combinations of various alternatives coupled with good management and husbandry practices will be the key to maximizing performance and maintaining animal productivity while we move forward with the ultimate goal of reducing the use of antibiotics in the animal agriculture industry.

## Figures and Tables

**Table 1:** Recombinant vaccines against coccidiosis in chickens.

Target Antigens	Source (*Eimeria* spp.)	Administration Route	Vectors	Immune Response or Effects on Chcikens	References
EF1-α/EF2 *	*E. acervulina*, *E. maxima*, *E. tenella*	Immunized subcutaneously	pcDNA3.1 (+)	Increased body weight gain, improved immune response, and decreased fecal oocyst shedding	[114,115]
SO7	*E. tenella*	Immunized intramuscularly	pcDNA3, pVR1012	Increased body weight gain, reduced oocyst shedding, and cecal lesion score	[118,119]
Gam82	*E. maxima*	Immunized intramuscularly	pET28a (+), pTRA-ERH	Improved immune responses, increased body weight gain, reduced oocyst shedding and gut pathology	[120,122,124]
Gam56	*E. maxima*	Immunized intramuscularly	pcDNA3.1(zeo)+	Improved immune responses, increased body weight gain, and decreased oocyst shedding	[120,123]
EtSAG4	*E. tenella*	Chest intramuscular injection	pET28a	Improved cell-mediated immunity, increased average body weight, and reduced oocyst output	[127]
α-tubulin	*E. acervulina*	Immunized subcutaneously	pGEM-T	Reduced duodenal lesions	[133]
GAPDH *	*E. acervulina*, *E. maxima* *E. tenella*	Immunized intramuscularly	pSDEP2AIMP1S	Improved immune response, reduced gut lesions, increased body weight gain, and decreased oocyst shedding	[114,116]
Em14-3-3 *	*E. maxima*	Immunized subcutaneously, oral immunization	pVAX1	Improved immune responses, decreased gut lesions, and increased body weight gain	[114,117]
IMP1	*E. maxima*	Oral immunization	pSDEP2AIMP1S, pGEMT	Increased body weight gain, reduced parasite replication and gut lesions	[112,135]
AMA1	*E. maxima*	Oral immunization	pSDEP2AIMP1S	Increased body weight gain, reduced *Eimeria* replication, and reduced gut lesions	[112,134]
Profilin (3-1E)	*E. acervulina*, *E. tenella*, *E. maxima*	in ovo immunization, immunized intramuscularly	pcDNA3.1 (+), pET32a (+), pSDEP2ARS,	Enhanced immunogenicity, increased body weight gain, and reduced gut pathology	[81,93,131,132,151]

* Indicates antigens that are common immunodominant proteins among *E. acervulina*, *E. tenella*, and *E. maxima*.

## Data Availability

Not applicable.
